# A Cohort Study on Deficiency of ADA2 from China

**DOI:** 10.1007/s10875-023-01432-8

**Published:** 2023-02-18

**Authors:** Guo-min Li, Xu Han, Ye Wu, Wei Wang, Hong-xia Tang, Mei-ping Lu, Xue-mei Tang, Yi Lin, Fan Deng, Jun Yang, Xin-ning Wang, Cong-cong Liu, Wen-jie Zheng, Bing-bing Wu, Fang Zhou, Hong Luo, Liang Zhang, Hai-mei Liu, Wan-zhen Guan, Shi-hao Wang, Pan-feng Tao, Tai-jie Jin, Ran Fang, Yuan Wu, Jie Zhang, Yao Zhang, Tian-nan Zhang, Wei Yin, Li Guo, Wen-jing Tang, Hong Chang, Qiu-ye Zhang, Xiao-zhong Li, Jian-guo Li, Zhi-xuan Zhou, Si-rui Yang, Kang-kang Yang, Hong Xu, Hong-mei Song, Natalie T. Deuitch, Pui Y. Lee, Qing Zhou, Li Sun

**Affiliations:** 1 National Children’s Medical Center, Shanghai, China; 2 Department of Rheumatology, Children’s Hospital of Fudan University, Shanghai, China; 3 Life Sciences Institute, Zhejiang University, Hangzhou, China; 4 Peking University First Hospital, Beijing, China; 5 Department of Pediatrics, Peking Union Medical College Hospital, Chinese Academy of Medical Sciences, Beijing, China; 6 Wuhan Children’s Hospital Tongji Medical College Huazhong University of Science & Technology, Wuhan, China; 7 Department of Rheumatology Immunology and Allergy, Children’s Hospital, Zhejiang University School of Medicine, Hangzhou, China; 8 Department of Rheumatology and Immunology, Children’s Hospital of Chongqing Medical University, Chongqing, China; 9 Affiliated Hospital of Qingdao University, Qingdao, China; 10 The Children’s Hospital of Soochow, Suzhou, China; 11 Department of Rheumatology and Immunology, Shenzhen Children’s Hospital, Shenzhen, China; 12 Affiliated Children’s Hospital of Capital Institute of Pediatrics, Beijing, China; 13 Division of Rheumatology, Immunology & Allergy in the Department of Pediatrics, The First Hospital of Jilin University, Changchun, China; 14 Department of Rheumatology, The Second Affiliated Hospital and Yuying Children’s Hospital of Wenzhou Medical University, Wenzhou, China; 15 Medical Transformation Centre, Children’s Hospital of Fudan University, Shanghai, China; 16 No. 960 Hospital of the Joint Service Support Force of the Chinese People’s Liberation Army, Jinan, China; 17 Department of Respiratory Medicine, The Second Xiangya Hospital, Central South University, Changsha, China; 18 Hunan Provincial People’s Hospital, Hunan, China; 19 National Human Genome Research Institute, Bethesda, MD USA; 20 Division of Immunology, Boston Children’s Hospital, Harvard Medical School, Boston, MA USA

**Keywords:** Adenosine deaminase 2, Deficiency of adenosine deaminase 2, Hematology, Hematopoietic stem cell transplantation, Vasculitis, TNF inhibitors

## Abstract

**Purpose:**

Deficiency of adenosine deaminase 2 (DADA2), an autosomal recessive autoinflammatory disorder caused by biallelic loss-of-function variants in adenosine deaminase 2 (ADA2), has not been systemically investigated in Chinese population yet. We aim to further characterize DADA2 cases in China.

**Methods:**

A retrospective analysis of patients with DADA2 identified through whole exome sequencing (WES) at seventeen rheumatology centers across China was conducted. Clinical characteristics, laboratory findings, genotype, and treatment response were analyzed.

**Results:**

Thirty patients with DADA2 were enrolled between January 2015 and December 2021. Adenosine deaminase 2 enzymatic activity was low in all tested cases to confirm pathogenicity. Median age of disease presentation was 4.3 years and the median age at diagnosis was 7.8 years. All but one patient presented during childhood and two subjects died from complications of their disease. The patients most commonly presented with systemic inflammation (92.9%), vasculitis (86.7%), and hypogammaglobinemia (73.3%) while one patient presented with bone marrow failure (BMF) with variable cytopenia. Twenty-three (76.7%) patients were treated with TNF inhibitors (TNFi), while two (6.7%) underwent hematopoietic stem cell transplantation (HSCT). They all achieved clinical remission. A total of thirty-nine *ADA2* causative variants were identified, six of which were novel.

**Conclusion:**

To establish early diagnosis and improve clinical outcomes, genetic screening and/or testing of ADA2 enzymatic activity should be performed in patients with suspected clinical features. TNFi is considered as first line treatment for those with vascular phenotypes. HSCT may be beneficial for those with hematological disease or in those who are refractory to TNFi.

**Supplementary Information:**

The online version contains supplementary material available at 10.1007/s10875-023-01432-8.

## Introduction

Deficiency of adenosine deaminase 2 (DADA2, OMIM 165,688) is an autosomal recessive autoinflammatory disease caused by biallelic loss-of-function variants in the *ADA2* gene, formerly named *CECR1* (cat eye syndrome chromosome region, candidate 1), located at chromosome 22q11.1 [1–3]. DADA2 was first described in 2014 by two separate groups in individuals with polyarteritis nodosa (PAN) and recurrent strokes [1, 2, 4]. However, the clinical spectrum of the disease has expanded considerably since its initial description.

DADA2 typically presents in childhood with systemic inflammation, vasculitis, humoral immunodeficiency, and/or hematologic abnormalities [1, 2, 5–13]. Inflammatory features of DADA2 can include recurrent fevers, mild to moderate anemia, and elevated inflammatory markers, such as erythrocyte sedimentation rate (ESR) and C-reactive protein (CRP) and musculoskeletal involvement (arthralgia/arthritis and/or myalgia/myositis). Vasculitis can involve in multiple organs, may manifest as cutaneous inflammation, early-onset ischemic (lacunar), and/or hemorrhagic strokes. Some patients may display humoral immunodeficiency of variable severity characterized by low immunoglobulin levels and increased risk of infection [1, 2, 5]. Hematologic symptoms are present in a subset of DADA2 patients and can include pure red cell aplasia (PRCA) that mimics Diamond-Blackfan anemia, lymphopenia, neutropenia, thrombocytopenia, or pancytopenia resulting in bone marrow failure (BMF) [6, 9, 14–18]. More severe manifestations of disease include organ damage, such as ischemic injury to the intestine, kidney, and/or digits, hepatosplenomegaly, and strokes. Sequelae of strokes can include progressive central neurologic deficits, ataxia, dysarthria, cranial nerve palsies, and cognitive impairment, which contributes to significant morbidity of this disease [1, 2, 12, 13, 19–23].

Adenosine deaminase 2 (ADA2) is an extracellular enzyme primarily secreted by myeloid cells [21, 24, 25]. ADA2 is a dimeric enzyme with four domains, including signal peptide, catalytic domain, putative receptor binding domain, and dimerization domain [11, 24, 26]. Biallelic variants in the *ADA2* gene result in decreased levels of ADA2 enzyme, which can be used as a marker of pathogenicity [1, 2]. Missense variants are most common, but nonsense variants, insertions/deletions (indels), splice-site variants, copy number variations (CNV), and structural variations have been described [11, 21, 26–29]

Early diagnosis and treatment initiation is essential for improving clinical outcomes and preventing more severe manifestation of disease. This study describes the clinical and genetic features in a Chinese cohort of thirty patients with DADA2 from seventeen centers in China. We discuss new clinical and genetic findings in this disease and summarize the current knowledge of DADA2 to help improve its recognition.

## Materials and Methods

### Study Design

This study was approved by ethics committees at Children’s Hospital of Fudan University, Shanghai, China, and was designed as a retrospective cohort study. The inclusion criteria were to have biallelic variants in the *ADA2* gene, plus at least one of the followings: (1) systemic inflammation, (2) vasculitis, (3) humoral immunodeficiency, (4) hematologic abnormalities, and (5) low level of ADA2 enzymatic activity. Patients were enrolled through a nationwide collaboration with approval by the local ethics committees. Research diagnostic testing was performed with written informed consent from the parent or patients (if more than 18 years of age).

### ADA2 Activity Detection

We assessed ADA2 enzyme activity in patients’ serum and cell cultures using a commercial kit for ADA2 enzyme activity (Diazyme Laboratories). Peripheral blood was collected and serum was separated by centrifugation. Enzyme activity was detected by plate reader (Synergy NEO2). ADA2 activity was isolated from total ADA activity by inhibiting ADA1 with erytro-9-(2-hydroxy-3-nonyl) adenine (EHNA, 1 mg/mL).

### Cell Preparation, Culture, and Transfection

Human embryonic kidney (HEK) 293 T cells were acquired from the American Type Culture Collection. HEK293T cells were grown in Dulbecco’s modified Eagle medium (Gibco) supplemented with 10% fetal bovine serum (FBS) (ExCell Bio) and penicillin/streptomycin (HyClone).

### Expression Plasmids

An ADA2 expression plasmid was constructed by cloning the *ADA2* coding sequence to pXC backbone. Other ADA2 mutant plasmids were constructed by introducing point mutation to the ADA2 expression plasmid with PCR.

### Western Blotting

Cells were lysed in cold cell lysis buffer (20 mM Tris–HCL, pH 7.4, 150 mM NaCl, 0.5% NP-40, and complete protease inhibitors) for 10 min and centrifuged at 20,000 g, 4 ℃ for 10 min. Protein concentration was measured on the cleared lysates by BCA protein assay kit (Thermo Fisher).

### DNA Sequencing

Genomic DNA was extracted and purified from peripheral whole-blood using a DNA isolation kit (Qiagen, Hilden, Germany). Targeted exome capture was conducted on the genomic DNA from each patient by using the SureSelect Human All Exon Target Enrichment System (Agilent). The captured exomes were sequenced using the Illumina HiSeq 2500 Sequencer platform (Illumina, San Diego, CA, USA). Whole exome sequencing (WES) and bioinformatic analysis were performed in patients and their families as previously described [30]. Variants identified by WES analysis were confirmed by Sanger sequencing. Variants were subsequently analyzed by various bioinformatics programs (SIFT, Polyphen2, PROVEAN, M-CAP, fathmm-MKL).

### RNA Sequencing

Libraries were prepared with one microgram of RNA using NEBNext Ultra RNA Library Prep Kit for Illumina (NEB) following the manufacturer’s instructions. Prepared libraries were sequenced on Illumina Novaseq platform and 150-bp pair-end reads were generated. Reads were mapped to human genome (GRCh38) with STAR (v2.7.10).

### Deletion Detection

Sequencing reads were visualized with IGV tools. Reads were colored in red or blue based on read strand. In one patient, absence of coverage for exon 7 paired with abnormal transcript could be observed directly in IGV indicating a deletion. The Alu element location on which the breakpoint of large deletion resided was detected by RepeatMasker (www.repeatmasker.org).

### Statistical Analysis

Statistical analyses were performed using the statistical package SPSS (Statistical Package for the Social Science; SPSS Inc., Chicago, IL, USA) version 22 and Microsoft Excel (Microsoft Office 2016 version 16.0; Microsoft Corporation, Redmond, WA, USA). Continuous variables are presented by the median and range, and categorical variables are presented as percentages and frequencies. The differences between two groups were analyzed by the Mann–Whitney *U* test, and chi-square test was used for comparison of categorical variables. A *p* value of ≤ 0.05 was considered significant.

## Results

### Clinical Characteristics

Between January 2015 and December 2021, thirty patients (ten female and twenty male) from families who met the inclusion criteria were recruited from seventeen centers in China. All but one subject were under the age of 18 years old. The median age at presentation was 4.3 years (range 9 days to 25.6 years), and median age at diagnosis was 7.8 years of age (range 1.0 to 30.0 years). Recurrent fever and rash were the first signs of disease in twenty-three patients (76.7%). Three patients (10.0%) presented with recurrent fevers alone and one (3.3%) presented with recurrent fever and respiratory tract infection. Systemic inflammation was noted in 93.3% of patients, defined by the presence of recurrent fever (90.0%), elevated ESR (66.7%), and/or elevated CRP (86.7%). The prevalence of hypogammaglobinemia in this cohort was 73.3%. The frequency of low levels of IgM, IgA, and IgG was 66.7%, 33.3%, and 26.7%, respectively (Table 1).

**Table 1:** Clinical feature of 30 patients with *ADA2* variants

Characteristic	
Male gender, *n* (%)	20 (66.7)
Children, *n* (%)	29 (96.7)
Median age at onset (*y*)	4.3
Median age at diagnosis (*y*)	7.8
Death *n* (%)	2 (6.7)
Clinical features, *n* (%) or no./total no	
Systemic inflammation	28 (93.3)
Recurrent fevers	27 (90.0)
Weight loss	10 (33.3)
Cutaneous involvement	26 (86.7)
Livedo racemosa/reticularis	18 (60.0)
Rash	18 (60.0)
Oral/skin ulcer	5 (16.7)
Erythema nodosum/nodosum	4 (13.3)
Raynaud phenomenon	4 (13.3)
Digital gangrene	1 (3.3)
Musculoskeletal system	13 (43.4)
Arthritis/arthragia	11 (36.7)
Muscle involvement	2 (6.7%)
Nervous involvement	18 (60.0)
Ischemic stroke	14 (46.7)
Hemorrhagic stroke	6 (20.0)
Central nervous system	17 (56.7)
Headache	3 (10.0)
Peripheral neuropathy	1 (3.3)
Ophthalmological findings	5 (16.7)
Central retinal artery occlusion	5 (16.7)
Gastrointestinal involvement	5 (16.7)
Intestinal hemorrhage	2 (6.7)
Intestinal perforation	2 (6.7)
Intestinal necrosis	1 (3.3)
Splenomegaly	5(16.7)
Hepatomegaly	1 (3.3)
Hepatosplenomegaly	7 (23.3)
Cardiovascular involvement	1 (3.3)
Hypertension	3 (10.0)
Immunodeficiency	22 (73.3)
Hypogammaglobulinemia	22 (73.3)
Recurrent infection	4 (13.3)
Hematologic abnormalities	22 (73.3)
Erythrocytopenia	22 (73.3)
Leukopenia/neutropenia	6 (20.0)
Lymphopenia	2 (6.7)
Pancytopenia	1 (3.3)
Laboratory findings, *n* (%) or no./total no	
Elevated erythrocyte sedimentation rate	20 (66.7)
Elevated C-reactive protein	26 (86.7)
Decreased hemoglobin	22 (73.3)
Low level IgM	20 (66.7)
Low level IgA	10 (33.3)
Low level IgG	8 (26.7)
Low ADA2 activity	15/15
Positive ANA	3 (10.0)
Skin biopsy	6
Polyarteritis nodosa	3 (50.0)
Vasculitis	2 (33.3)
Panniculitis	1 (16.7)

Systemic inflammation is defined as one of the followings, recurrent fever, weight loss, elevated ESR, or elevated CRP; weight loss is defined as less than average weight of the same age and sex reduce two standard deviation (X-2SD)

86.7% of patients had cutaneous manifestations. Livedo racemosa/reticularis (LR, 60.0%) and non-specific erythematous rash (60.0%) were the most common features of cutaneous involvement. 60.0% of patients had some form of neurologic manifestations. 46.7% of patients had experienced at least one ischemic stroke, and 20.0% had a history of hemorrhagic stroke. Hepatosplenomegaly was present in 23.3% of patients, with isolated splenomegaly in 16.7% of patients and isolated hepatomegaly in 3.3% of patients. Other common features included arthritis/arthralgia in 36.7% of patients, gastrointestinal tract abnormalities in 16.7%, and eye involvement (central retinal artery occlusion) in 16.7%. Hypertension was noted in 10.0% patients. Less common features included myocarditis, muscle weakness, and myositis (Table 1).

Twenty-four patients were first diagnosed by rheumatologists. The remainder were diagnosed by neurology (*n* = 4, 13.3%), pulmonary medicine (*n* = 1, 3.3%), and hematology (*n* = 1, 3.3%). All patients underwent clinical and laboratory investigations in one of seventeen centers, including genetic evaluation. The clinical and laboratory characteristics of the patients are provided in Table S1 and S2. Summarized data for the cohort are presented in Table 1. Consanguinity was noted in three families. 12 patients had previously been reported in the literature [31–34].

### Novel Pathogenic Variants in DADA2

All suspicious variants in *ADA2* were identified by WES and confirmed by Sanger sequencing. Databases including gnomAD, ExAC, 1000 genomes, HGMD, and Infevers were used to assess frequency of the variants and only those found in < 1% of the population were included. Missense variants were assessed for their potential to disrupt protein function using SIFT, Polyphen2, PROVEAN, M-CAP, and fathmm-MKL (Table 2).

**Table 2:** Genetic information in patients with *ADA2* variants

Case	Genomic change^1^	cDNA change^2^	Amino acid Substitution	Novel variant	Variant status^3^	Parental consanguinity^4^	Computational prediction^5^
1	g.17181578 T > C	c.1443-2A > G	-	-	Hom	Y	….D
2	g.17209536C > T	c.142G > A	p.G48R	-	Het	N	DDDDN
	g.17209357delT	c.321delA	p.A109Lfs*11	Novel	Het		….
3	g.17182626 T > A	c.1217A > T	p.E406V	-	Het	N	DDDDD
	g.17209578G > A	c.100C > T	p.R34W	-	Het		DDTDN
4	g.17209534delC	c.144delG	p.R49Gfs*4	-	Het	Y	….
	g.17203738G > A	c.578C > T	p.P193L	-	Het		DDDDD
5	g.17209539C > G	c.139G > C	p.G47R	-	Hom	Y	DDDDD
6	g.17209539C > G	c.139G > C	p.G47R	-	Het	N	DDDDD
	g.17188355G > T	c.1065C > A	p.F355L	-	Het		TPDDD
7	g.17156950_17215337del	-	-	-	Het	N	….
	g.17187845_17188621del	-	-	-	Het		….
8	g.17209539C > A	c.139G > T	p.G47W	-	Het	N	DDDDD
	g.17207129A > G	c.484 T > C	p.W162R	Novel	Het		DDDDD
9	g.17191715A > C	c.849 T > G	p.F283L	Novel	Hom	N	DDDDD
10	g.17189998G > A	c.916C > T	p.R306X	-	Het	N	….N
	g.17188351C > T	c.1069G > A	p.A357T	-	Het		DDDDD
11	g.17181904 T > C	c.1358A > G	p.Y453C	-	Hom	N	DDDDD
12	g.17203745delG	c.571delC	p.Q191Sfs*5	-	Hom	N	….
13	g.17181925A > G	c.1337 T > C	p.F446S	-	Het	Y	DDDDD
	g.17182022C > T	c.1240G > A	p.V414M	-	Het		DDDDD
14	g.17209536C > T	c.142G > A	p.G48R	-	Het	N	DDDDN
	g.17188348C > T	c.1072G > A	p.G358R	-	Het		DDDDD
15	g.17207233 T > A	c.380A > T	p.N127I	-	Het	N	DDDDD
	g.17188443C > A	c.977G > T	p.G326V	-	Het		DDDDD
16	g.17188416 T > G	c.1004A > C	p.H335P	-	Het	N	TBDDD
	g.17207224 T > C	c.389A > G	p.Y130C	-	Het		DDDDD
17	g.17209539C > G	c.139G > C	p.G47R	-	Het	N	TPNTN
	-	Exon 7	-	-	Het		-
18	g.17207107C > T	c.506G > A	p.R169Q	-	Het	N	DDDDD
	g.17207220delC	c.393delG	p.R131Sfs*53	-	Het		….
19	g.17181904 T > C	c.1358A > G	p.Y453C	-	Het	N	DDDDD
	g.17182734 T > A	c.1109A > T	p.N370I	-	Het		DDDDD
20	g.17209536C > T	c.142G > A	p.G48R	-	Het	N	DDDDN
	g.17207108G > C	c.505C > G	p.R169G	-	Het		DDDDN
21	g.17209536C > T	c.142G > A	p.G48R	-	Het	N	DDDDN
	g.17207108G > C	c.505C > G	p.R169G	-	Het		DDDDN
22	g.17209534_17209535delinsA	c.143_144delinsT	p.G48Vfs*5	-	Het	N	….
	g.17209581dupT	c.97dupA	p.T33Nfs*29	-	Het		….
23	g.17209581dupT	c.97dupA	p.T33Nfs*29	-	Het	Y	….
	g.17188348C > T	c.1072G > A	p.G358R	-	Het		DDDDD
24	g.17181955A > G	c.1307 T > C	p.M436T	Novel	Het	N	DDDDD
	g.17181928A > G	c.1334 T > C	p.M445T	Novel	Het		TBNDD
25	g.17209539C > A	c.139G > T	p.G47W	-	Het	N	DDDDD
	g.17207108G > C	c.505C > G	p.R169G	-	Het		DDDDN
26	g.17209538C > A	c.140G > T	p.G47V	-	Het	N	….
	g.17182768A > T	c.1082-7 T > A	-	-	Het		….
27	g.17181904 T > C	c.1358A > G	p.Y453C	-	Het	N	DDDDD
	g.17182768A > T	c.1082-7 T > A	-	-	Het		….
28	g.17209539C > A	c.139G > T	p.G47W	-	Hom	Y	DDDDD
29	g.17209581dupT	c.97dupA	p.T33Nfs*29	-	Het	N	….
	g.17207267delT	c.346delA	p.I116Lfs*4	Novel	Het		….
30	g.17189980G > A	c.934C > T	p.R312X	-	Het	N	….N
	g.17209400A > G	c.278 T > C	p. I93T	-	Het	N	DDDDD

^1^gDNA reference: NC_000022. ^2^cDNA reference: NM_001282225. ^3^*Hom*, homozygous; *Het*, heterozygous. ^4^*Y*, yes; *N*, no. ^5^Computational prediction: letters are on behalf of the prediction of SIFT, Polyphen2, PROVEAN, M-CAP, and fathmm-MKL in turn. *D*, damage; *B*, benign; *T*, tolerant; *N*, neutral; *P*, possibly damaging; *dot*, no prediction

Among the 30 patients, 24 were found to have compound heterozygous variants while six had homozygous variants (Table 2). One patient carried two deletions (g.17156950_17215337del and g.17187845_17188621del), which were inherited *in trans* (Table 2 and Supplementary Fig. 1a-d). These two deletions were detected by visualizing the raw RNA-seq and WES data using IGV (Supplementary Fig. 2). The larger deletion is a 58 kb deletion spanned from *HDHD5* exon1 to *ADA2* exon2 (Supplementary Fig. 1e) and the smaller deletion was restricted to *ADA2* exon7 (Supplementary Fig. 1f). These likely represent Alu-mediated deletions.

**Fig. 1:**
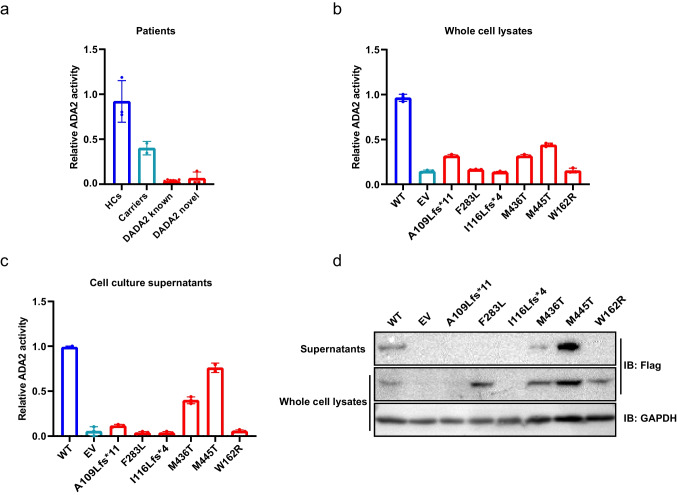
ADA2 activity of novel *ADA2* variants measured in patients and cell cultures. (**a**) ADA2 activity of DADA2 patients carrying novel *ADA2* variants is lower than carriers with known *ADA2* pathogenic variants (*n* = 2) or HC (*n* = 3). (**b**, **c**) ADA2 activity of whole cell lysates and supernatants of 293 T cells transfected with novel ADA2 mutants compared to WT. (**d**) Western blots of 293 T cells transfected with novel ADA2 mutants. As P9 has gone and P2 is lost to follow up, ADA2 activity of these patients was not been tested. HC, healthy control; WT, wild type; EV, empty vector

In total, we identified six novel deleterious *ADA2* variants in this cohort (Table 2). Plasma or serum ADA2 activity of patients carrying novel *ADA2* variants was tested for enzymatic activity. All tested patients exhibited lower ADA2 enzymatic activity when compared to healthy (*n* = 3) and carrier (*n* = 2) controls (Fig. 1a). We also transfected plasmids of these novel ADA2 mutants into HEK293T cells and tested ADA2 activity in the supernatants and cell lysates. Enzymatic activity was reduced in both whole cell lysates and supernatants of cell cultures in these constructs when compared to wild-type ADA2 (Fig. 1b–d). These data support the deleterious nature of these variants. Although there are six novel variants found in our cohort, these patients with novel variants have no unique phenotype (Table S1).

### Treatment and Outcome

The median follow-up duration was 20.2 months (range 5 to 36 months) after the diagnosis of DADA2.

Two patients (6.7%) died from macrophage activation syndrome (MAS), characterized by fever, splenomegaly, multi-lineage cytopenia, hyperferritinemia, hypertriglyceridemia, and hypofibrinogenemia. Although they were both treated with pulse methylprednisolone combined with cyclosporine, they developed multiple organ failure leading to eventual death.

Before being diagnosed with DADA2, twenty-seven (90.0%) patients were treated with high-dose glucocorticoids, and six (20.0%) patients received non-steroidal anti-inflammatory drugs (NSAIDs) (Table 3 and Table S3). In total, twenty-six (86.7%) patients received one or more traditional disease-modifying anti-rheumatic drugs (DMARDs), including methotrexate (*n* = 7, 23.3%), cyclosporine (*n* = 5, 16.7%), mycophenolate mofetil (*n* = 5, 16.7%), cyclophosphamide (*n* = 4, 13.3%), hydroxychloroquine (*n* = 3, 10.0%), and sulfasalazine (*n* = 1, 3.3%). Eleven patients (36.7%) received intravenous immunoglobulin (IVIG), and five (16.7%) were given thalidomide. In addition, four patients (13.3%) were treated with tocilizumab. Except for thalidomide, the overall efficacy of these interventions was suboptimal, as patients continued to have significant symptoms while on therapy. After being diagnosed with DADA2, twenty-three (76.7%) patients were switched to TNFi, including etanercept (*n* = 13, 43.3%), infliximab (*n* = 1, 3.3%), and adalimumab (*n* = 9, 30.0%). TNFi was significantly reduced fever episodes, vasculitis, and no patients have had a stroke during the time they have been on treatment.

**Table 3:** Therapy for patients with DADA2

Therapy	Medicine	Patient, *n* (%)	The efficacy
Traditional DMARDs	Sulfasalazine	1 (3.3)	Little effect
	Methotrexate	7 (23.3)	Little effect
	Hydroxychloroquine	3 (10.0)	Little effect
	Cyclophosphamide	4 (13.3)	Little effect
	Tacrolimus	1 (3.3)	Little effect
	Cyclosporine	5 (16.7)	Little effect
	Mycophenolate	5 (16.7)	Little effect
Biological DMARDs	Tocilizumab	4 (13.3)	Failure to control inflammation
	Etanercept	13 (43.3)	Controlling the fever episodes, vasculopathy, and prevention of strokes
	Infliximab	1 (3.3)	
	Adalimumab	9 (30.0)	
Others	Glucocorticoid	27 (90.0)	Little effect
	IVIG	11 (36.7)	Little effect
	NSAIDs	6 (20.0)	Little effect
	Thalidomide	5 (16.7)	Controlling the fever episodes, vasculopathy, and prevention of strokes
	HSCT	2 (6.7)	Control both the immunological, the hematological, and the vascular phenotype of DADA2

*DMARDs*, disease-modifying anti-rheumatic drugs; *IVIG*, intravenous immunoglobulin; *HSCT*, hematopoietic stem cell transplantation; *NSAIDs*, non-steroidal anti-inflammatory drugs

Two patients (6.7%) received hematopoietic stem cell transplantation (HSCT). The first had presented with recurrent fever and rash and then developed gangrene of the fingers before 5 months old [32]. This patient was refractory to glucocorticoid and tocilizumab treatment and further developed right central retinal artery occlusion. Given her lack of response to treatment and severe organ involvement, she underwent HSCT, which was successful. The second patient had bone marrow failure (BMF) with variable cytopenia and was referred for HSCT after failing trials of both glucocorticoids and cyclosporine.

## Discussion

In this report, we describe the clinical features, laboratory findings, genotypes, and treatment responses in thirty Chinese patients with DADA2. This is the largest cohort study on DADA2 from China to date. The mortality of DADA2 in this cohort is 6.7%, which is in line with rates in other reported cohorts [35–40]. Vasculitis with variable organ involvement and systemic inflammation was noted in majority of patients, and CRP appears to be a more sensitive index for inflammation compared to ESR.

DADA2 is increasingly being recognized as a monogenic etiology for PAN with systemic inflammation and vasculitis features and biallelic variants in *ADA2* have been identified in ~ 25–31% of childhood PAN cases [1, 2, 8, 11, 21, 26, 28, 38–42]. Genetic testing and/or ADA2 activity detection should be considered in all patients with recurrent fever accompanied unexplained elevated CRP and/or ESR, especially in those with livedo racemosa/reticularis and evidence of PAN-like vasculitis.

Though the prevalence of hypogammaglobinemia in DADA2 patients is high, immunodeficiency, with recurrent or severe infections, is relatively rare [1, 2, 6, 8, 10, 12, 35–39]. There were seven patients (23.3%) with recurrent infections in this cohort, most of which had mild respiratory tract infections that resolved with minimal intervention. Interestingly, one patient (P28) developed recurrent fever and respiratory tract infection without and cutaneous or vascular symptoms. However, at 15 years old, he developed variable immunodeficiency with low levels of IgM, IgA, and IgG. Mild humoral immunodeficiency with low immunoglobulin levels appears to be a common clinical feature of DADA2, regardless of the initial phenotype [11, 28, 40]. This suggests that DADA2 screening should be considered in the differential diagnosis for patients with unexplained antibody deficiencies.

In some patients, the primary manifestation of DADA2 appears to be hematologic abnormalities, without the vasculitis or systemic inflammation that was first identified as a hallmark of this disease [6, 9]. BMF with variable cytopenia was observed in one patient who is without vasculitis and systemic inflammation in this cohorts. Mild to moderate anemia and leukopenia/neutropenia were noted in 73.0% and 20% of patients, respectively. The frequency of anemia in this cohort, which may be caused by chronic systemic inflammation, is consistent with that of recurrent fever (90.0%).

**Fig. 2:**
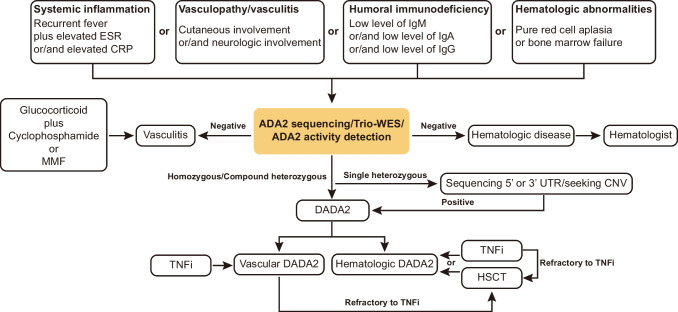
Flow chart of diagnosis and treatment of DADA2. HSCT, hematopoietic stem cell transplantation; MMF, mycophenolate mofetil; TNFi, TNF inhibitor

Most patients in this cohort were followed and characterized by rheumatologists. While the majority of features of this disease is rheumatologic in nature, it is possible that these findings were biased as symptoms including hematological abnormalities, humoral immunodeficiencies, and/or early stroke, which may be underrecognized by such providers [1, 2, 6, 8, 10, 12, 35–39, 43]. Furthermore, there is likely ascertainment bias in the patients who are identified by rheumatology, as opposed to another specialty who may recognize other elements of this multifaceted disease. It is imperative that providers outside of rheumatology (i.e., hematologists, immunologists, and neurologists) also learn to recognize DADA2, as early diagnosis and treatment can change the narrative of this condition. To facilitate the evaluation of individuals with possible DADA2, we propose the following schema for evaluation (Fig. 2). Diagnostic testing for DADA2 is recommended for patients with at least one of following manifestations: unexplained reason of systemic inflammation, vasculitis, humoral immunodeficiency, or severe hematologic abnormalities, especially in children. In the future, we hope that consensus clinical guidelines will formalize further steps in the evaluation, monitoring and treatment of individuals with or suspected to have DADA2.

In total, thirty-nine unique, deleterious variants in *ADA2* were detected in this cohort. Among these variants, six were novel (Table 2), expanding the spectrum of known pathogenic variants in *ADA2*. These novel variants may represent founder variants within the Chinese population. However, these patients with novel variant had classic features of DADA2 without any unique phenotype (Table S1). Variants detected in this cohort encompassed each coding exon of the *ADA2* gene and involved all four domains of the ADA2 protein. We did not observe any hot spots for variants in this cohort. The most common variant is the frameshift variant p.T33Nfs*29, which was identified in four unrelated patients. Several known variants were detected in this cohort including: p.G47R which is frequently seen in Georgian Jewish and Turkish cohorts, p.R169Q which is mainly noted in individuals from the Netherlands, Belgium, and Finland, and the p.T360A which has previously been reported in those from Italy [2, 8, 36, 44]. Further research may be able to classify where these variants first emerged and how specific they truly are to specific ancestral populations.

In terms of treatment, patients in this cohort showed no response to glucocorticoids, NSAIDs, IVIG, and traditional DMARDs. TNFi was used in twenty-three patients with active disease and was found to effectively control fever episodes and vasculitis. In addition, no patients treated with TNFi develop strokes or disease relapse during the follow-up period which ranged from 5 to 36 months. Three patients did respond to thalidomide therapy. HSCT is an alternative choice for patients with severe hematologic manifestation of disease or disease that is refractory to TNFi. The two patients who received HSCT in this cohort successfully achieved remission and are doing well. For these patients, HSCT not only rescued the hematological and immunological phenotypes but also appears to have been curative for their vascular phenotype [9, 10, 39, 45–48].

## Conclusion

DADA2 is a heterogeneous disease characterized by systemic inflammation, vasculitis, immunodeficiency, and hematologic manifestations. This work represents the largest cohort study of DADA2 patients in China to date. We identified six novel pathogenic *ADA2* variants. Based on our experience, we propose a set of criteria to facilitate the timely diagnosis of patients with DADA2 that encompasses the different disease phenotypes.

## Supplementary Information

Below is the link to the electronic supplementary material.

Supplementary file1 (DOCX 398 KB)

## Data Availability

The original contributions generated for the study are included in the article and Supplementary Material. Data in this article is available on GSA-Human (https://ngdc.cncb.ac.cn/gsa-human), accession number: HRA001673.
