# Supplementary material for: A Cohort Study on Deficiency of ADA2 from China
Source: J Clin Immunol. 2023 Feb 18;43(4):835–45. doi: 10.1007/s10875-023-01432-8 (PMC10110724; doi:10.1007/s10875-023-01432-8)
Supplement: Supplementary file 1 — Supplementary file1 (DOCX 398 KB) [file 10875_2023_1432_MOESM1_ESM.docx]

**Supplementary material**

Supplementary Appendix

Table S1: Characteristics of 30 patients with DADA2………………………………………………………………………………… Pg. 2

Table S2: Laboratory finding of 30 patients with ADA2 variants……………………………………………………………………...Pg. 5

Table S3: Treatment in 30 patients with ADA2 variants………………………………………………………………………………Pg. 7

Figure S1: Large fragment deletion analysis in patient’s family………………………………………………………………………...Pg. 8

Figure S2: Integrative Genomics Viewer (IGV) visualization of deletions………………………………………………………….…Pg.10

Table S1 Characteristics of 30 patients with DADA2

| Case | G | Age  onset  (y) | Age of  Diagnosis  (y) | Inflammation | | Vasculopathy (systemic involvement) | | | | | | Immunodeficiency | | Bone Marrow Failure | | Genotype |
| --- | --- | --- | --- | --- | --- | --- | --- | --- | --- | --- | --- | --- | --- | --- | --- | --- |
|  |  |  |  | Fever | WL | Cutaneous | Arthritis/  arthragia | Nervous | GI | Renal | Others | HGGN | Recurrent  infection | Hemo-  cytopenia | Hepatomegaly/  splenomegaly |  |
| 1 | F | 4.8 | 11.5 | Y | Y | LR and rash, RP,  oral and genital  ulcer, and DH | Arthritis | HS and IS | N | N | Hypertention | IgM | N | N | Both | c.1443-2A>G |
| 2 | M | 4.9 | 7.7 | Y | N | LR and rash,  oral ulcer | Arthritis | CRAO  IS | N | N | N | IgM | Y | N | Both | c.142G>A  c.321delA |
| 3 | M | 10.7 | 11.7 | Y | N | LR | N | IS | N | N | N | IgM | N | N | N | c.1217A>T  c.100C>T |
| 4 | M | 4.9 | 5.4 | Y | Y | LR | Arthragia | Peripheral  neuropathy | Vomit,  Diarrhea | N | N | IgM | N | N | Splenomegaly | c.144delG  c.578C>T |
| 5 | M | 5.3 | 15.6 | Y | Y | LR, rash and  oral ulcer | N | N | Colitis,  IH, IP, IN | N | Hypertention | N | N | N | Both | c.139G>C |
| 6 | M | 12.0 | 14.2 | N | N | LR and rash | Arthragia | N | N | N | N | N | N | N | N | c.139G>C  c.1065C>A |
| 7 | M | 25.6 | 30.0 | Y | Y | LR and rash | N | CRAO  HS | N | N | Headache | IgG  IgM  IgA | N | N | Splenomegaly | g.17156950_  17215337del  g.17187845_  17188621del |
| 8 | F | 2.3 | 6.2 | Y | Y | LR, rash, RP,EN | N | IS | N | N | Muscle  weakness | IgM  IgA | N | N | N | c.139G>T  c.484T>C |
| 9 | M | 0.1 | 12.9 | Y | Y | N | Arthritis | N | N | N | Myocarditis | IgM  IgG  IgA | Y |  | Splenomegaly | c.849T>G |
| 10 | M | 0.2 | 1.0 | Y | N | Rash | Arthritis | IS | IH，IP | N | Y | N | N | N | Both | c.916C>T  c.1069G>A |
| 11 | M | 8.0 | 14.0 | Y | N | EN, Purpura,  Skin ulcer | N | N | N | N | N | IgG  IgM  IgA | N | N | Splenomegaly | c.1358A>G |
| 12 | F | 7.7 | 12.1 | Y | N | EN, LR | Arthragia | CRAO  IS | N | N | LEP | IgM,  IgA | N | N | N | c.571delC |
| 13 | F | 0.2 | 1.2 | Y | N | LR, rash, DG | N | CRAO  IS | N | N | N | IgG  IgM | N | N | N | c.1337T>C  c.1240G>A |
| 14 | M | 4.4 | 8.1 | Y | N | LR, rash, RP | Arthritis | CRAO  IS | N | N | Myositis | IgG | N | N | N | c.142G>A  c.1072G>A |
| 15 | F | 1.0 | 5.0 | Y | N | LR, EN,  oral ulcer | Arthritis | HS and IS | Colitis | N | N | IgG  IgM  IgA | N | N | Hepatomegaly | c.380A>T  c.977G>T |
| 16 | M | 0.2 | 6.0 | Y | N | Rash | N | N | N | N | Hypertention | IgM | N | N | N | c.1004A>C  c.389A>G |
| 17 | M | 9.0 | 16.0 | Y | N | Rash | N | N | AP | N | N | IgM | N | N | N | c.139G>C  Exon 7 Del |
| 18 | M | 0.5 | 1.0 | Y | Y | LR | N | N | N | N | N | N | N | N | N | c.506G>A  c.393delG |
| 19 | F | 3.0 | 6.0 | Y | N | Rash | N | N | N | N | N | IgM | N | N | N | c.1358A>G  c.1109A>T |
| 20 | M | 0.1 | 8.5 | Y | N | LR | N | IS and HS | N | N | N | IgM | N | N | N | c.142G>A  c.505C>G |
| 21 | F | 3.5 | 4.2 | N | N | LR | N | N | N | N | N | N | N | N | N | c.142G>A  c.505C>G |
| 22 | M | 3.5 | 3.8 | Y | N | Rash | Arthragia | HS | N | N | N | IgM  IgA | N | Y | Both | c.143_144delinsT  c.97dupA |
| 23 | M | 6.9 | 7.0 | Y | N | N | N | N | N | N | N | IgG  IgA | N | N | Splenomegaly | c.97dupA  c.1072G>A |
| 24 | M | 6.8 | 6.9 | N | N | N | N | N | N | N | N |  | Y | Y | N | c.1307T>C  c.1334T>C |
| 25 | M | 4.2 | 6.0 | Y | N | EN | N | N | N | N | N | IgM | N | N | Both | c.139G>T  c.505C>G |
| 26 | F | 3.5 | 12.1 | Y | Y | Rash, LR | Arthritis | IS | N | N | N |  | Y | N | N | c.140G>T  c.1082-7T>A |
| 27 | F | 2.3 | 2.5 | Y | N | Rash, LR | N | IS | N | N | Headache | IgM  IgA | N | N | N | c.1358A>G  c.1082-7T>A |
| 28 | M | 15.0 | 24.7 | Y | N | N | N | IS | N | N | Headache | IgM | Y | N | Both | c.139G>T |
| 29 | F | 7.1 | 7.6 | Y | Y | Rash, RP, | N | IS, HS | N | N | LEP | IgM  IgA  IgG | y | N | N | c.97dupA  c.346delA |
| 30 | M | 0.5 | 7.1.0 | Y | Y | Rash | N | IS | N | N | N | N | y | Y | N | c.934C>T  c.278T>C |

*: Presence of family history, his sister died from vasculitis; Red letter: initial symptoms

AP: abdominal pain, CRAO: central retinal artery occlusion, DG: digital gangrene, DH: dermal hemangioma, EN: Erythema nodosum, G: Gender, GI: gastrointestinal, HGGN: hypogammaglobulinemia, HS: Hemorrhagic stroke, IH: intestinal hemorrhage, IN: intestinal necrosis, IP: intestinal perforation, IS: ischemic stroke, LEP: lower extremities pain, LR: Livedo reticularis/racemosa,N: No, RP: Raynaud phenomenon, Y: yes, WL: Weight loss.

Table S2 Laboratory finding of 30 patients with ADA2 variants

| Case | Complete blood count | | | **Pre-therapy** | | **Post-therapy** | | Immunoglobulins | | | Complement | | Lymphocyte subgroup count | | | | | ADA2  activity | TNF-α | ANA | Skin  biopsy |
| --- | --- | --- | --- | --- | --- | --- | --- | --- | --- | --- | --- | --- | --- | --- | --- | --- | --- | --- | --- | --- | --- |
|  | Leucocytes  10^9^/L | Hb  g/L | Platelets  10^9^/L | ESR  mm/h | CRP  mg/L | ESR  mm/h | CRP  mg/L | IgG  g/L | IgA  g/L | IgM  g/L | C3  g/L | C4  g/L | CD56^+^  /μL | CD19^+^  /μL | CD3^+^  /μL | CD4^+^  /μL | CD8^+^  /μL |  |  |  |  |
| 1 | 9.3 | 119 | 247 | 88.0 | 50.0 | 18 | <8 | 5.0 | 0.45 | 0.5 | 1.54 | 0.50 | 45.6 | 356.4 | 975.9 | 407.4 | 486.0 | - | Nor | N | - |
| 2 | 8.3 | 108 | 311 | 44.0 | 60.0 | 16 | <8 | 8.9 | 0.77 | 0.15 | 1.76 | 0.46 | 117.8 | 259.4 | 1403.7 | 763.6 | 540.4 | - | - | P | - |
| 3 | 8.3 | 124 | 347 | 40.0 | 18.0 | 6 | 6.2 | 7.8 | 0.94 | 0.26 | 1.57 | 0.43 | 210.5 | 251.4 | 1263.0 | 407.4 | 686.0 | - | H | N | - |
| 4 | 8.7 | 126 | 339 | 51.0 | 33.4 | 10 | 4.4 | 5.9 | 1.1 | 0.29 | 1.58 | 0.55 | 23.7 | 238.7 | 1756.8 | 835.5 | 876.4 | - | H | N | PAN |
| 5 | 8.9 | 107 | 327 | 22 | 49 | 16 | 9.1 | 14.9 | 3.43 | 0.53 | 1.46 | 0.25 | 57.7 | 581.7 | 1330.3 | 593.5 | 681.2 | - | Nor | N | PAN |
| 6 | 7.3 | 113 | 407 | 19 | 1.21 | 13 | <8 | 14.37 | 2.7 | 1.46 | 1.73 | 0.44 | 296 | 361 | 1330 | 588 | 479 | Low | - | N | PAN |
| 7 | 4.6 | 107 | 200 | 7 | 22.7 | 9 | <8 | 3.57 | 0.43 | 0.12 | 1.3 | 0.3 | 42.0 | 62.0 | 706.0 | 423.0 | 252.0 | Low |  |  |  |
| 8 | 6.3 | 107 | 286 | 26 | 40.4 | 11 | 8.6 | 7.83 | 0.26 | 0.18 | 1.19 | 0.3 | 121 | 307 | 1013 | 615 | 291 | Low | Nor | N | Vasculitis |
| 9 | 4.38 | 92 | 195 | 61.0 | 99.1 | - | - | 2.46 | 0.24 | 0.18 | 1.56 | 0.28 | 79 | 130 | 1180 | 621 | 532 | Low | Nor | N | - |
| 10 | 7.7 | 86 | 901 | 44 | 33 | 5 | <8 | 17.5 | 0.24 | 0.68 | 1.74 | 0.4 | 1098 | 2407 | 3781 | 2770 | 1205 | Low | Nor | N | - |
| 11 | 5.36 | 96 | 242 | 18.0 | 33.1 | 13 | <8 | 5.15 | 0.49 | 0.29 | 0.33 | 1.49 | 96.6 | 306.6 | 1167.4 | 618.7 | 387.7 | - | - | N | Panniculitis |
| 12 | 3.75 | 105 | 150 | 50.0 | 14.7 | 10 | <8 | 6.16 | 0.79 | 0.38 | 0.13 | 1.37 | 17.7 | 127.5 | 697.0 | 223.3 | 466.1 | - | Nor | P | - |
| 13 | 10.7 | 90 | 448 | 32 | 59.5 | 12 | <8 | 3.2 |  | 0.2 |  |  | 659.9 | 847.4 | 1657.1 | 961.4 | 590.4 | - | Nor | N | - |
| 14 | 7.0 | 90 | 784 | 16 | 59.0 | 16 | <8 | 6.39 | 0.43 | 1.32 | 1.41 | 0.3 | 10.4 | 11.0 | 51.0 | 27.0 | 19.0 | - | H | N | - |
| 15 | 8.2 | 84 | 309 | 74 | 44.3 | 8 | <8 | 5.1 | 0.28 | 0.27 | 1.35 | 0.148 | 129 | 164 | 1673 | 978 | 626 | Low | Nor | N | - |
| 16 | 9.1 | 120 | 429 | 95 | 80 | 10 | <8 | 1.92 | 0.31 | 017 | - | - | 50 | 213 | 864 | 242 | 242 | Low | - | P | - |
| 17 | 4.8 | 124 | 118 | 6 | 11 | 8 | <8 | 6.84 | 0.95 | 0.23 | 1.37 | 0.30 | 49 | 168 | 791 | 291 | 418 | Low | Nor | N | - |
| 18 | 21 | 98 | 132 | 49 | 43 | 10 | <8 | 6.95 | 0.66 | 1.07 | - | - | 803 | 5090 | 1722 | 1096 | 579 | Low | Nor | N | - |
| 19 | 4.1 | 122 | 224 | 9 | 3 | 1 | <8 | 5.33 | 0.46 | 0.31 | 1.07 | 0.30 | 80 | 336 | 1007 | 603 | 310 | - | - | N | - |
| 20 | 7.4 | 96 | 239 | 18 | 32.5 | 6 | 7.2 | 9.2 | 1.5 | 0.15 | 1.40 | 0.57 | 10.2 | 13.5 | 76.8 | 42.0 | 29.1 | Low | Nor | N | - |
| 21 | 8.0 | 103 | 334 | 49 | 14.1 | 12 | <8 | 18.2 | 2.36 | 0.60 | 1.659 | 0.56 | 8.1 | 24.1 | 63.0 | 39.9 | 19.4 | Low | Nor | N | - |
| 22 | 1.2 | 77 | 121 | 99 | 15 | 9 | <8 | 3.6 | 0.067 | 0.08 | 1.18 | 0.37 | 44.60 | 113.67 | 985.73 | 471.9 | 458.8 | - | Nor | N | Vasculitis |
| *23* | 1.5 | 112 | 229 | 16 | 36.2 | 18 | <8 | 5.34 | 0.30 | 0.69 | 1.19 | 0.33 | 3.1 | 120 | 597 | 337 | 219 | - | Nor | N | - |
| 24 | 1.73 | 53 | 56 | 12 | <8 | 10 | <8 | 28.5 | 1.45 | 0.69 | 1.15 | 0.4 | 234.4 | 327.1 | 912.0 | 327.1 | 355.8 | Low | - | N | - |
| 25 | 7.4 | 99 | 299 | 77 | 111.3 | 16 | <8 | 12.7 | 2.58 | 0.36 | 1.737 | 0.56 | - | - | - | - | - | - | - | - | - |
| 26 | 9.52 | 133 | 355 | 37 | 11 | 9 | <8 | - | - | - | - | - | - | - | - | - | - | - | - | N | - |
| 27 | 14,4 | 62 | 459 | 55 | 92.2 | 11 | 5.1 | - | 0.16 | 0.43 | - | - | - | - | - | - | - | Low | - | - | - |
| 28 | 2.18 | 99 | 99 | 14 | 6 | - | - | 3.12 | 0.07 | 0.07 | 0.82 | 0.22 | - | - | - | - | - | - | - | - | - |
| 29 | 8.45 | 96 | 213 | 44 | 65 | 12 | 6.1 | 5.06 | 0.66 | 0.3 | 2.23 | 0.44 | 3.03 | 85.8 | 1282 | 815 | 383 | Low | - | - | - |
| 30 | 2.25 | 98 | 99 | 25 | 104 | 8 | <8 | 9.06 | 0.32 | 0.20 | 1.67 | 0.32 | - | - | - | - | - | Low | - | - | - |

The red font indicates higher than the normal reference value, while the blue font indicates lower than the normal reference value

N: negative, P: positive, PAN: polyarteritis nodosa, Hb: Hemoglobin

Table S3 Treatment in 30 patients with ADA2 variants

| Case | Previous treatment | | | | | | | | | | | | Current treatment | | | | | death |
| --- | --- | --- | --- | --- | --- | --- | --- | --- | --- | --- | --- | --- | --- | --- | --- | --- | --- | --- |
|  | 1NSAIDs | 2GC | 3MTX | 4SSZ | 5Thalidomide | 6HCQ | 7CTX | 8MMF | 9CSA | 10IVIG | 11TCZ | Efficacy | Etanercept | Infliximab | Adalimumab | Others | Efficacy |  |
| P1 | Y | Y | Y | Y | Y | N | N | N | N | N | N | NR | N | N | Y | 1+2+3+5 | CR | N |
| P 2 | N | Y | N | N | N | Y | N | Y | N | N | N | NR | Y | N | N | 2+6+7 | CR | N |
| P 3 | N | Y | N | N | N | N | N | N | N | N | N | NR | Y | N | N | 2 | CR | N |
| P 4 | N | Y | N | N | N | N | N | Y | N | N | N | NR | Y | N | N | 2+8 | CR | N |
| P 5 | Y | Y | Y | N | N | N | N | N | Y | Y | N | NR | N | N | Y | N | PR | N |
| P 6 | N | Y | N | N | N | N | N | N | N | N | N | NR | Y | N | N | N | PR | N |
| P 7 | N | Y | N | N | N | N | N | N | N | N | N | NR | Y | N | N | N | CR |  |
| P 8 | N | Y | N | N | N | N | N | N | N | N | N | NR | N | N | Y | 2 | PR | N |
| P9 | N | Y | N | N | N | N | N | N | N | Y | N | NR | - | - | - | - | - | Y |
| P10 | Y | Y | Y | N | N | N | N | N | Y | Y | Y | PR | N | N | Y | 2+FK506 | CR | N |
| P11 | Y | Y | N | N | Y | Y | Y | Y | N | Y | N | PR | N | N | N | 1+5+10 | CR | N |
| P12 | N | Y | Y | N | Y | N | Y | Y | N | Y | N | PR | N | N | Y | 5+10 | CR | N |
| P13 | N | Y | N | N | N | N | N | N | N | N | Y | NR | N | N | N | HSCT | CR | N |
| P14 | N | Y | N | N | N | N | Y | N | N | Y | N | NR | Y | N | N | 2 | CR | N |
| P15 | N | Y | Y | N | Y | N | N | N | Y | Y | Y | NR | Y | N | N | 2+3+5 | CR | N |
| P16 | N | N | N | N | N | N | N | N | Y | Y | N | NR | N | N | N | 3 | CR | N |
| P17 | N | Y | Y | N | N | Y | N | N | N | N | N | NR | N | N | Y | 8 | CR | N |
| P18 | N | Y | N | N | N | N | N | N | N | Y | N | NR | N | N | Y |  | CR | N |
| P19 | N | N | N | N | N | N | N | N | N | N | N | NR | N | N | N | 1 | CR | N |
| P20 | N | Y | N | N | N | N | N | N | N | Y | N | NR | Y | N | N | N | CR | N |
| P21 | N | N | N | N | N | N | N | N | N | N | N | - | Y | N | N |  | CR | N |
| P22 | Y | Y | N | N | N | N | N | N | N | N | Y | NR | N | Y | N |  | CR | N |
| *P23* | N | Y | Y | N | N | N | N | N | N | N | N | PR | N | N | Y | 2+3 | CR | N |
| P24 | N | Y | N | N | N | N | N | N | Y | N | N | NR | N | N | N | HSCT | CR | N |
| P25 | N | Y | N | N | N | N | N | N | N | N | N | NR | Y | N | N | 1 | CR | N |
| P26 | N | Y | N | N | N | N | N | N | N | Y | N | NR | Y | N | N | N | CR | N |
| P27 | N | Y | N | N | N | N | N | N | N | Y | N | NR | Y | N | N | N | CR | N |
| P28 | N | Y | N | N | N | N | N | N | N | N | N | NR | N | N | N | N | - | Y |
| P29 | Y | Y | N | N | Y | N | Y | Y | N | N | N | NR | N | N | Y | N | CR | N |
| P30 | N | Y | N | N | N | N | N | N | N | Y | N | NR | Y | N | N | N | CR | N |

MHSCT: Myeloablative hematopoietic stem cell transplantation, TCZ: Tocilizumab, NSAID, Non steroidal anti.inflammatory drugs; AZT, azathioprine; CTX, cyclophosphamide; CyA, ciclosporin A; MMP, mycophenolate mofetil; MTX, methotrexate, SSZ: Sulfasalazine.

CR：complete remission, Persistent control of inflammatory parameters with no disease’s flares or complications in the absence of any steroid treatment; PR：partial remission, good control of disease activity with sporadic relapses and need of steroid on demand or increased steroid dosage; NR: no remission poor, little or absent response with persistence of systemic flares and/or complication.


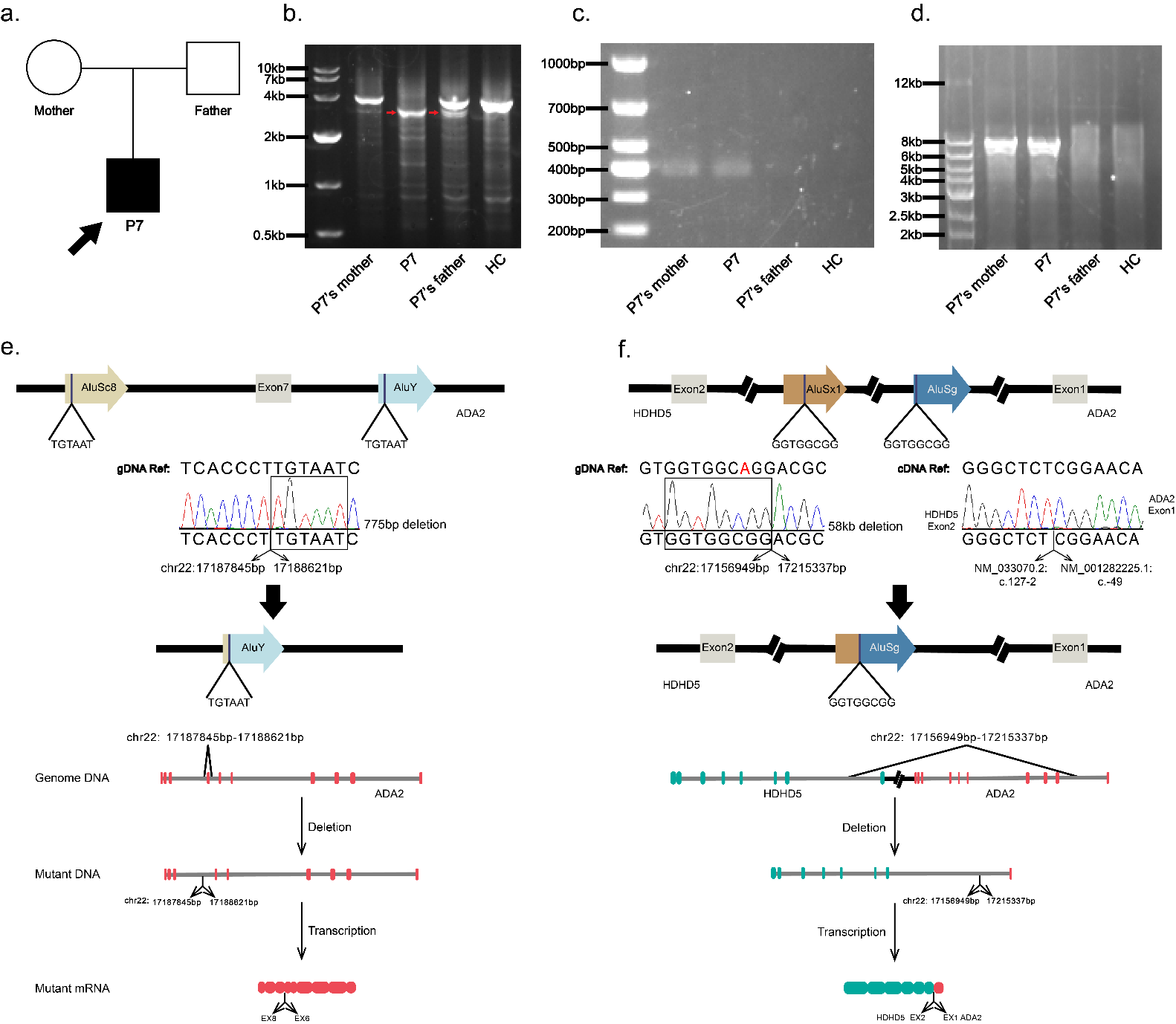


**Supplementary Fig. 1** Identification of long-range deletions in P7’s family. **a** Pedigree of P7’s family. **b** PCR using patient’s gDNA with a set of primer flanking ADA2 exon 7 shows a homozygous deletion and a heterozygous deletion in P7 and P7’s father, respectively. **c** PCR using patient’s cDNA with a set of primer flanking a region from HDHD5 exon2 to ADA2 exon1, indicating a fusion transcript. **d** Long-range PCR using patient’s gDNA with a set of primer flanking a region from HDHD5 exon1 to ADA2 exon2, indicating a deletion of about 58kb in P7 and P7’s mother. **e,f** Schematic demonstration of Alu-mediated deletion of exon7 and of a long region from HDHD5 exon1 to ADA2 exon2.


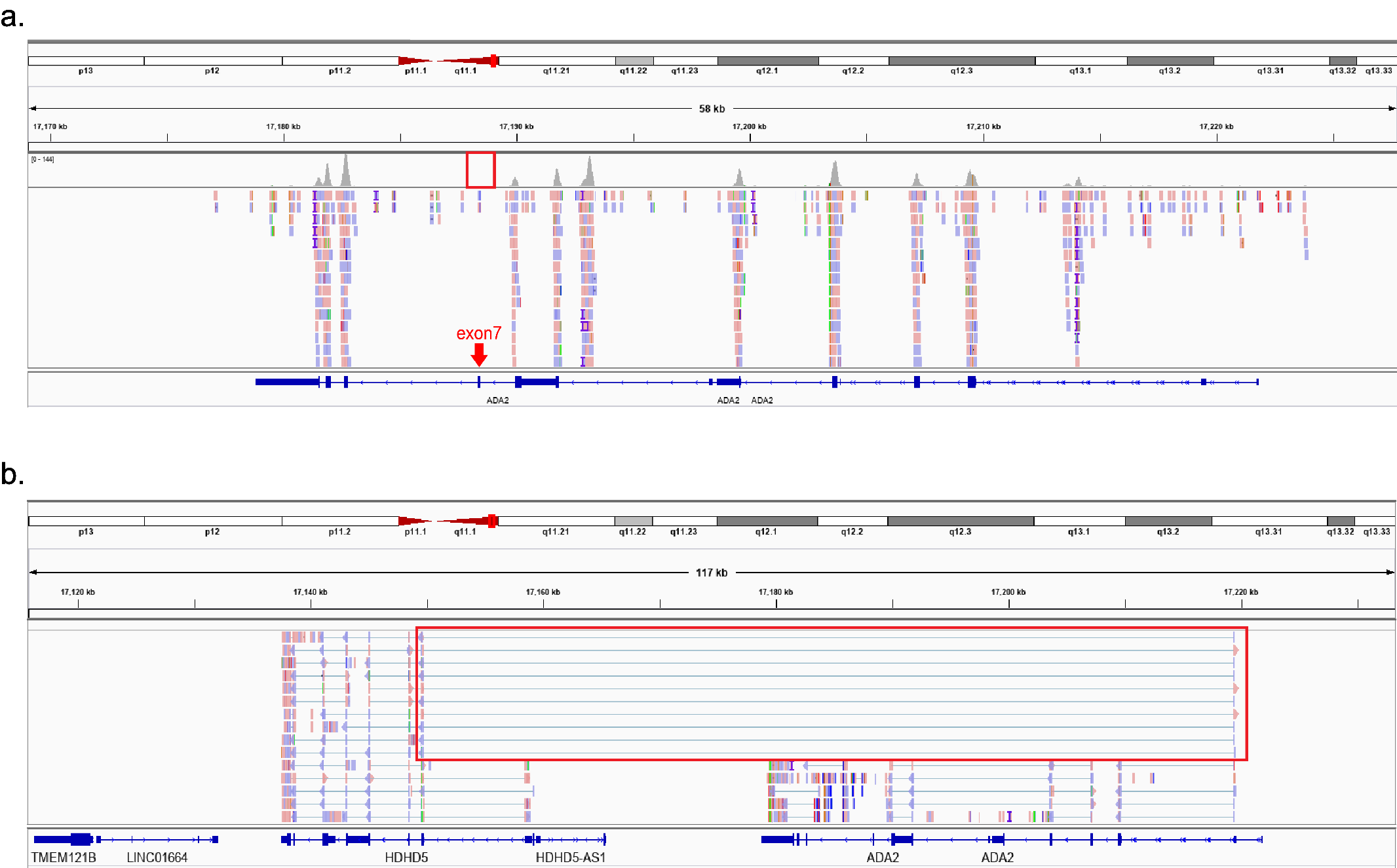


**Supplementary Fig. 2** View of deletions of P7 in IGV. **a** Excerpt of WES data visualized with IGV shows a deletion of ADA2 exon 7 in P7. **b** Excerpt of RNAseq data visualized with IGV shows abnormal splicing of P7’s ADA2 transcripts.
